# Anti‐biofilm and anti‐virulence effects of silica oxide nanoparticle–conjugation of lectin purified from *Pseudomonas aeruginosa*

**DOI:** 10.1049/nbt2.12022

**Published:** 2021-03-03

**Authors:** Sahira Nsayef Muslim, Alaa Naseer Mohammed Ali, Ibtesam Ghadban Auda

**Affiliations:** ^1^ Biology Department Mustansiriyah University Baghdad Iraq

## Abstract

*Pseudomonas aeruginosa* lectin is purified and nanoparticle‐conjugated in an attempt to inhibit biofilm formation. Thirteen (23.6%) *P. aeruginosa* isolates are obtained from chicken meat samples, of which 30.8% are biofilm producers and 69.2% are lectin producers. Lectin is purified 36.8‐fold to final specific activity of 506.9 U/mg. Four nanoparticle types are prepared via laser ablation: platinum (Pt), gold (Au), silica oxide (SiO_2_), and tin oxide (SnO_2_). The four types are characterised, and pulse feeding is used to conjugate the lectin and nanoparticles. Pt, Au, SiO_2,_ and SnO_2_ nanoparticles inhibit biofilm formation, especially SiO_2_ nanoparticles, which have higher effectiveness when conjugated with purified lectin. SiO_2_‐conjugated lectin significantly (*p* < 0.05) inhibits biofilm formation more effectively than control and other nanoparticle‐conjugated lectins. Au‐, Pt nanoparticle‐, and SnO_2_‐conjugated lectins inhibit biofilm significantly compared with control (*p* < 0.05), and *rhlR* gene expression is decreased in the presence of SiO_2_‐conjugated lectin. Furthermore, lectin and Pt, Au, SiO_2_ and SnO_2_ nanoparticles separately, and their conjugated lectins, are effective biofilm inhibitors. Of these, SiO_2_‐conjugated lectin was most significant as an anti‐biofilm. Moreover, virulence factors regulon and RhlR were reduced by SiO_2_‐conjugated lectin, indicating that this conjugation may also decrease the virulence of *P. aeruginosa*.

## 1 INTRODUCTION

Biological and chemical contamination can originate from foods of animal origin [1, 2]. The resources of bacterial contamination are water, fodder, the environment, and other animals. Bacteria grown on chicken meat can cause different types of infections as consumed by humans as well as food poisoning [1, 3]. *Pseudomonas*, *Acinetobacter*, *Escherichia*, *Flavobacterium* and *Salmonella* species predominate and exist on poultry skin, but the internal organs are usually sterilised. Viruses, bacteria, and mould can contaminate chicken meat during processing [1, 2, 4]. Animal foods are naturally contaminated by contaminants of feathers, skin, the respiratory tract, and the reproductive and digestive systems. Furthermore, airborne as well as water‐, sewer‐, and soil‐based pathogens can contaminate foods of animal origin. The amounts and types of microbes on foods depend largely on processing sanitation [5]. Lectins are multivalent or divalent carbohydrate‐binding proteins that can precipitate glycoconjugates and polysaccharides or agglutinated cells [6]. Hemagglutinins and lectins are various groups of non‐immune glycoproteins or proteins that have more than one non‐catalytic part reversibly bound to specific oligosaccharides or monosaccharides [7]. Hemagglutinins and lectins occur in viruses, bacteria, plants, and animals. They have chemical properties that make them a helpful tool in numerous fields such as cell biology, immunology, membrane structure, molecular biology, genetic engineering, pharmacology, clinical chemistry, and cancer research [8]. The term nanoparticles refers to any particle whose three dimensions are measured on the nanoscale of atoms or molecules [9]. Metal nanoparticles such as those consisting of copper, gold (Au), and silver (Ag) are an interesting nanoparticles owing to their novel magnetic, chemical, electrical, optical, and physical properties [10, 11]. The Au nanoparticle has many applications and has unique chemical and physical properties [12, 13]. A formidable gelatinous biofilm is formed by the bacteria that is capable of adhering to virtually any surfaces [14]. Biofilms in burn and cystic fibrosis patients lead to serious skin and lung infections and may lead to death. Furthermore, biofilms can cause ear infections, sinusitis, gum disease, and tooth decay [15]. The exopolysaccharides matrix (EPS) of biofilms consists of nucleotides, polysaccharides, and proteins. Dissolution of biofilm EPS by enzymes such as EPS depolymerises may be the most effective way to eliminate biofilm [16]. In industry, biofilms are challenging because they cause corrosion in pipelines for water distribution and oil transfer. Moreover, biofilms constitute treatment failures, as they are responsible for antibiotic resistance [14, 15]. Therefore, this study attempts to isolate *Pseudomonas aeruginosa* from chicken meat, testing its ability to form biofilm and produce lectin. Furthermore, lectin is purified and conjugated with specific nanoparticles to investigate its anti‐biofilm activity against biofilm‐forming *P. aeruginosa*.

## 2 MATERIALS AND METHODS

### 2.1 Samples and the identification of bacterial isolates

One gram from 55 samples of chicken carcasses was homogenized in 100 ml of 0.85% NaCl solution. One milliliter of the solution was inoculated on blood and MacConkey agars. The pure culture isolates were initially identified by biochemical characteristics such as oxidase and catalase [17]. *P. aeruginosa* isolates were finally identified by the Vitek 2 system (bioMérieux, France). After *P. aeruginosa*–suspected isolates were isolated and purified, the inoculum (with 0.5 to 0.6 McFarland turbidity tubes) was inoculated into the GN VITEK 2 cassette used for Gram‐negative bacteria, and the identification card was placed in the neighbouring slot. The incubation and reading of card results was system‐performed.

### 2.2 Genotypic identification

The *rpsL* gene (*16S rRNA* gene) was used for genotypic identification of *P. aeruginosa*. The bacterial genomic DNA was isolated by the boiling method [18] and amplified by the PCR technique. The oligonucleotide primers of the *rpsL* gene used for detection of *P. aeruginosa* are the forward primer 5′‐ CAAGCGCATGGTCGACAAG‐3′ and the reverse primer 5′‐ GCTGTGCTCTTGCAGGTTG‐3′, yielding an amplicon of 201 bp. The DNA products were visualised by gel electrophoresis [19].

### 2.3 Detection of *Pseudomonas aeruginosa* lectin producers

#### 2.3.1 Semi‐quantitative screening

The detection of *P. aeruginosa* lectin production by semi‐quantitative analysis was performed according to work by Eshdat and Sharon (1982). The formation of agglutination within 5 min indicates a positive result. A mixture of red blood cells with phosphate buffer saline (PBS) without *P. aeruginosa* suspension served as a control [20].

#### 2.3.2 Quantitative haemagglutination screening

The *P. aeruginosa* isolate culture or lectin suspension serial twofold dilution in microtitre plates was dispensed with an equal volume of 3% O^+^ human red blood cells and then incubated at 37°C for 120 min. The activity was expressed in haemagglutination units (H.U.). One H.U. was defined as the inverse of the highest dilution still capable of causing agglutination [21].

### 2.4 Extraction and purification of lectin

The selected *P. aeruginosa* isolate was cultivated on the colonisation factor antigen medium [22], then incubated at 30^o^C/24 h, the cells were harvested by the centrifugation, washed twice, and re‐suspended in 0.02 M PBS, pH 7.2. The cells were disrupted by glass beads at 4°C for 50 min with the aid of vortexing. Cell debris was excluded by centrifugation for 20 min at 8000 rpm. The supernatant was used to measure haemagglutination activity of the resultant crude lectin extracts. Ammonium sulphate was used for the precipitation of the protein, the Sephadex G‐200 column (Sigma, St., Louis, USA) was used to purify the lectin, and concentration and haemagglutination activity was estimated. Transmission electron microscopy (TEM) was used to determine the shape of the purified lectin before and after conjugation with nanoparticles.

### 2.5 Protein content estimation

Lectin protein content was determined by the Bradford method [23]. The method is based on increased absorption from 465 to 595 nm due to binding of Coomassie Brilliant Blue G‐250 to the protein.

### 2.6 Preparation of the nanoparticles

Four types of nanoparticles, namely, platinum (Pt), Au, silica oxide (SiO_2_), and tin oxide (SnO_2)_, were prepared by laser ablation as described elsewhere [9]. The four nanoparticles were prepared using four pure metal pellets (99.99%). The pellets were pressed to reach a thickness of 2 mm. The neodymium‐doped yttrium aluminium garnet laser at 1 Hz repetition with number shots of laser fourteen pulses (flounce 14.45 J/cm 2) and wavelength of 1064 nm was operated to create the four nanoparticles.

### 2.7 Characterisation of nanoparticles

For confirmation of colloidal nanoparticle formation and the characterisation of the four nanoparticles, the following techniques were applied: X‐ray diffraction in the Department of Physics/College of Science/Baghdad University, UV‐visible spectrophotometer with a wavelength ranging from 300 to 900 nm in the Department of Physics/College of Science/Mustansiriyah University, and TEM in Al‐Mansoura University/Egypt.

### 2.8 Biofilm formation

Two methods of biofilm formation were used, Congo red agar (CRA) plate and polystyrene plate, for the biofilm formation assay.

#### 2.8.1 Congo red agar plate assay

Biofilm formation was screened by CRA plate assay as described by the method of Freeman et al. [24]. The test isolates were cultivated on the CRA plates and incubated overnight at 37°C and 25°C. The result was considered positive if rough black colonies appeared on the agar plates.

#### 2.8.2 Polystyrene plate for biofilm formation assay

To detect the biofilm formation ability of *P. aeruginosa* isolates, the polystyrene plate method was used and the results were interpreted as described elsewhere [25]. A *P. aeruginosa* suspension at 10^6^ CFU ml^−1^ was prepared and placed in a 96‐well polystyrene plate, and each plate was incubated at 37°C for one day. The plates were then stained with 0.4% crystal violet; absorbance was measured at 570 nm. The plates with cultured media without bacteria served as blanks.

#### 2.8.3 Biofilm inhibition assay

SiO_2_‐conjugated lectin was prepared by the method of feeding and pulses [9]. Briefly, the nanoparticles and prepared lectin (1.01 μg/ml) were mixed at 4^o^C and pulsed by laser at a 1 Hz repetition rate with 40 pulses, fluency of 14.44 J/cm^2^, and a wavelength of 1064 nm. The biofilm inhibition capability of the above nanoparticles was also measured. One milliliter of the conjugated lectin was used to inhibit biofilm by the CRA and polystyrene plate methods. The plates were inoculated with *P. aeruginosa* and incubated overnight. The percentage of inhibition of biofilm was calculated by the following equation:

%inhibition=[(ControlOD570nm−TestOD570nm)/ControlOD570nm×100][24].

### 2.9 Expression of quorum‐sensing regulatory gene in the presence of lectin and silca oxide nanoparticles

Quorum‐sensing (QS) regulatory gene (*rhlR* gene) expression was measured by real‐time RT–PCR after and before treatment with the lectin–SiO_2_ nanoparticles that exhibited the best antibacterial ability. SiO_2_ nanoparticles and lectin at 1.01 ng/ml of purified lectin were used. Late log phase culture of *P. aeruginosa* was used for RNA extraction by the RNeasy kit (Qiagen Inc., South Korea). The *rpsL* (*16S rRNA* gene‐above primer set) served as the normalising gene. The primers for *rhlR* expression were 5ʹ‐GACCAGGAGTTCGACCAGTT‐3ʹ and 5ʹ‐GGTAGGCGAAGACTTCCTTG‐3ʹ, and the probe was 5ʹ‐CCGACGACCGACGCCCGAC CT‐3ʹ. The Brilliant QRT–PCR Master Mix (Stratagene, California, USA) kit was used for the gene expression experiments. The samples were run three times [26].

### 2.10 Statistical analysis

Data were analysed by ANOVA at *p* < 0.05 significance.

## 3 RESULTS

### 3.1 Isolation of *Pseudomonas aeruginosa*

**FIGURE 1:**
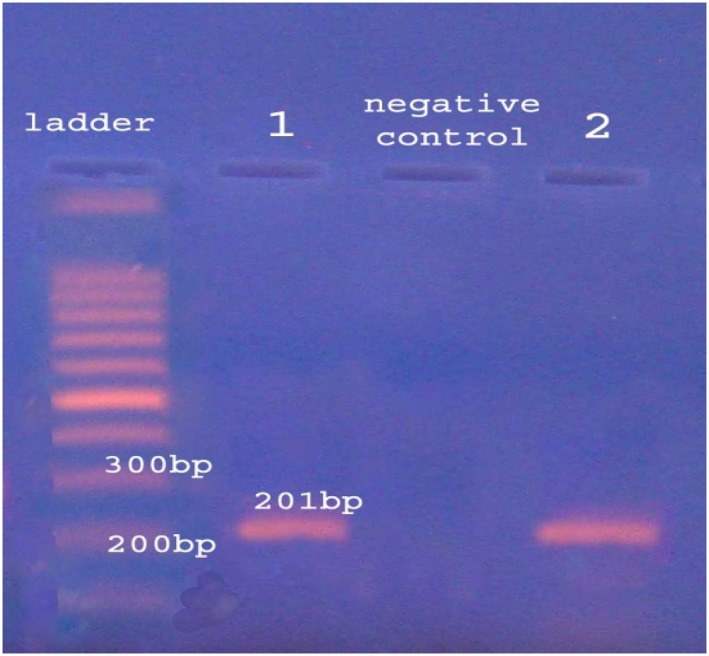
*Pseudomonas aeruginosa* identified by *rpsL* gene amplification

Thirteen (23.6%) *P. aeruginosa* isolates were obtained from 55 samples from chicken carcasses. Phenotypic identification of *P. aeruginosa* was done by the Vitek 2 system and confirmed by genotypic recognition relying upon certain housekeeping amplification (*rpsL*) as shown in Figure 1.

The agarose gel electrophoresis of *rpsL* gene amplicon of *P. aeruginosa*. Lane M: 100 bp DNA ladder, lanes 1 and 2 are positive results with 201 bp amplicon and negative control. The electrophoresis was run in 1.5% agarose gel, 7 V/cm for 90 min.

### 3.2 Detection of lectin producers

**FIGURE 2:**
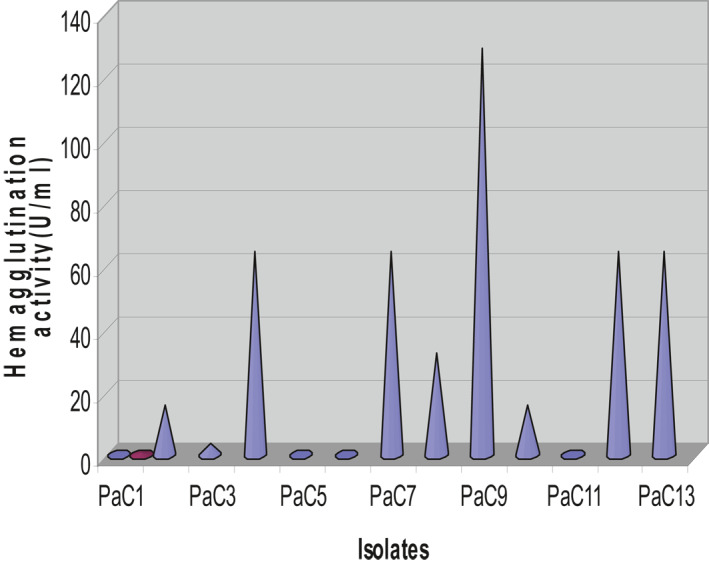
Quantitative analysis of haemagglutination activity of *Pseudomonas aeruginosa* isolates

Haemagglutination activities in a glass slide and microtitre plate revealed that 9 (69.2%) of 13 *P. aeruginosa* isolates showed haemagglutination activity. *P. aeruginosa* C9 showed a higher haemagglutination value and reached a higher titre (128 U/ml), as shown in Figure 2.

### 3.3 Extraction and purification of lectin

**FIGURE 3:**
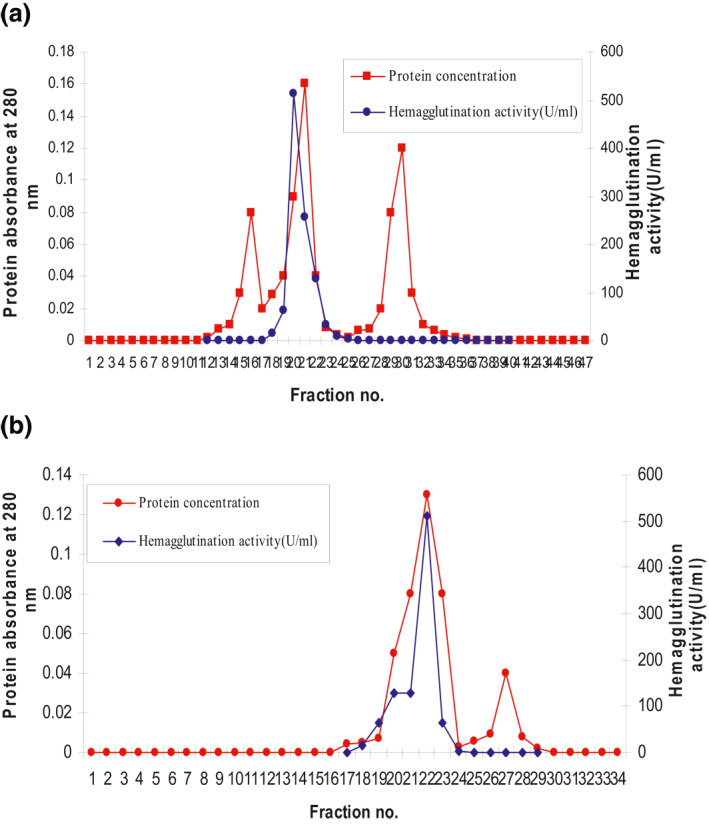
(a) Purification of *Pseudomonas aeruginosa* C_9_ lectin by ion‐exchange chromatography. (b) Purification of *P. aeruginosa* C_9_ lectin by gel filtration chromatography

First, lectin was extracted from C9 *P. aeruginosa* isolate, and haemagglutination activity was raised from 128 to 256 U/ml, and specific activity was 62.43 U/mg. The extracted lectin subjected to the first step of purification included precipitation of lectin by ammonium sulphate at 60% saturation as reported in Table 1. After centrifugation, the supernatant was loaded on the ion‐exchange column. At this step, lectin was purified 29.7‐fold with a yield of 50% using a sodium chloride gradient. The activity was at the second peak (Figure 3a). The purification was accomplished by gel filtration. Lectin was purified to 36.8‐fold and a yield of 40%, obtaining a final specific activity of 506.9 U/mg, the highest activity detected in the first peak (Figure 3b). Lectin was purified with a yield of 72.72% and 40.76‐fold of purification.

**TABLE 1:** Steps of lectin purification from *Pseudomonas aeruginosa* C_9_

The purification step	Haemagglutination activity (U/ml)	Protein concentration (mg/ml)	Specific activity (U/mg)	Total activity (U/mg)	Purification fold
Crude extract	128	9.3	13.76	5120	1
(NH_4_)_2_SO_4_ precipitation	256	4.10	62.43	3840	4.5
QAE Sephadex	512	1.25	409.6	2560	29.7
Sephadex G‐200	512	1.01	506.9	2048	36.8

### 3.4 Nanoparticle preparation and characterisation

#### 3.4.1 Ultraviolet‐visible spectrum

**FIGURE 4:**
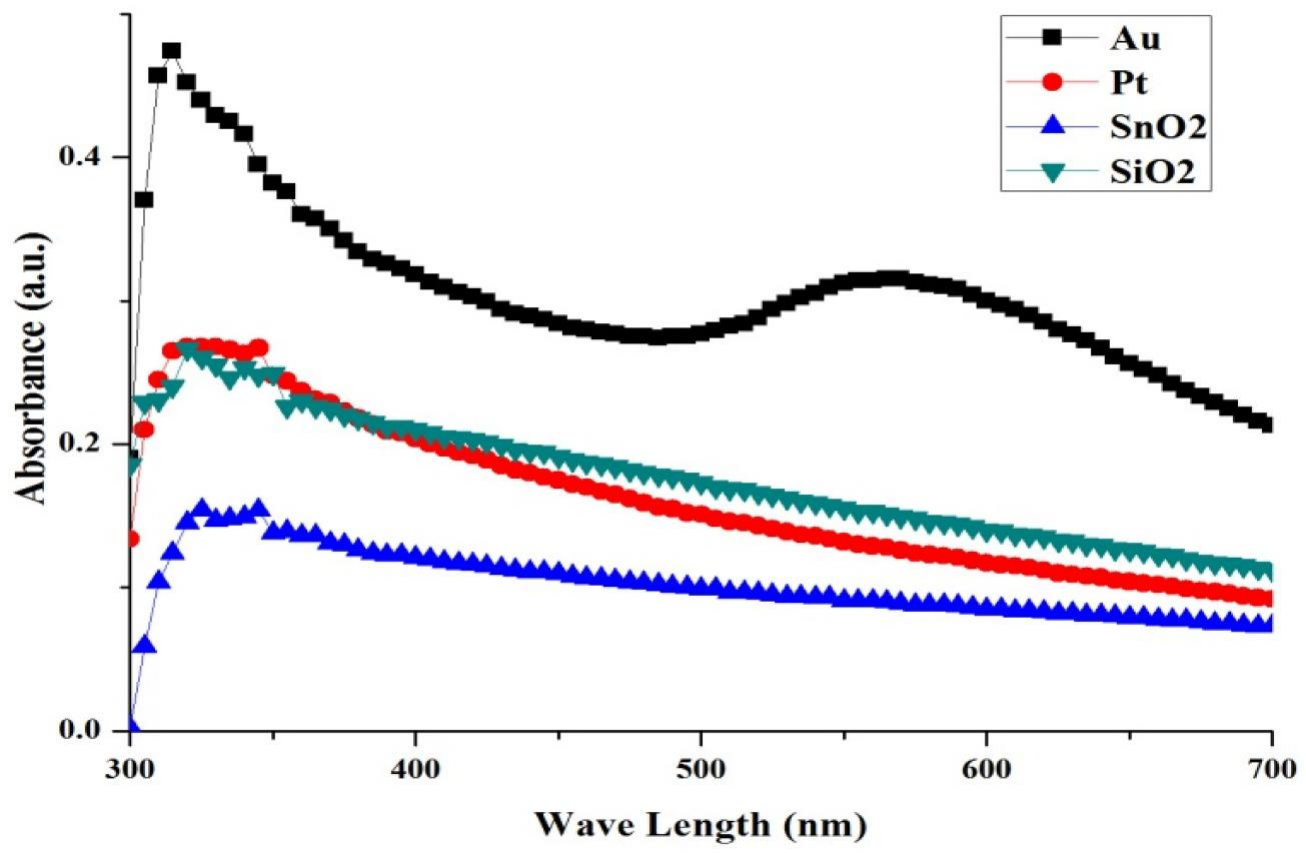
Absorbance of Pt, Au, SiO_2_, and SnO_2_ nanoparticle thin films. Au, gold; Pt, platinum; SnO_2_, tin oxide; SiO_2_, silica oxide

The variation of the absorbance (A) with the wavelength (nm) for the nanoparticle thin films is shown in Figure 4. The Pt, Au, SiO_2_, and SnO_2_ sizes increased with increases in the spectra of the absorption.

#### 3.4.2 X‐ray diffraction investigation

**FIGURE 5:**
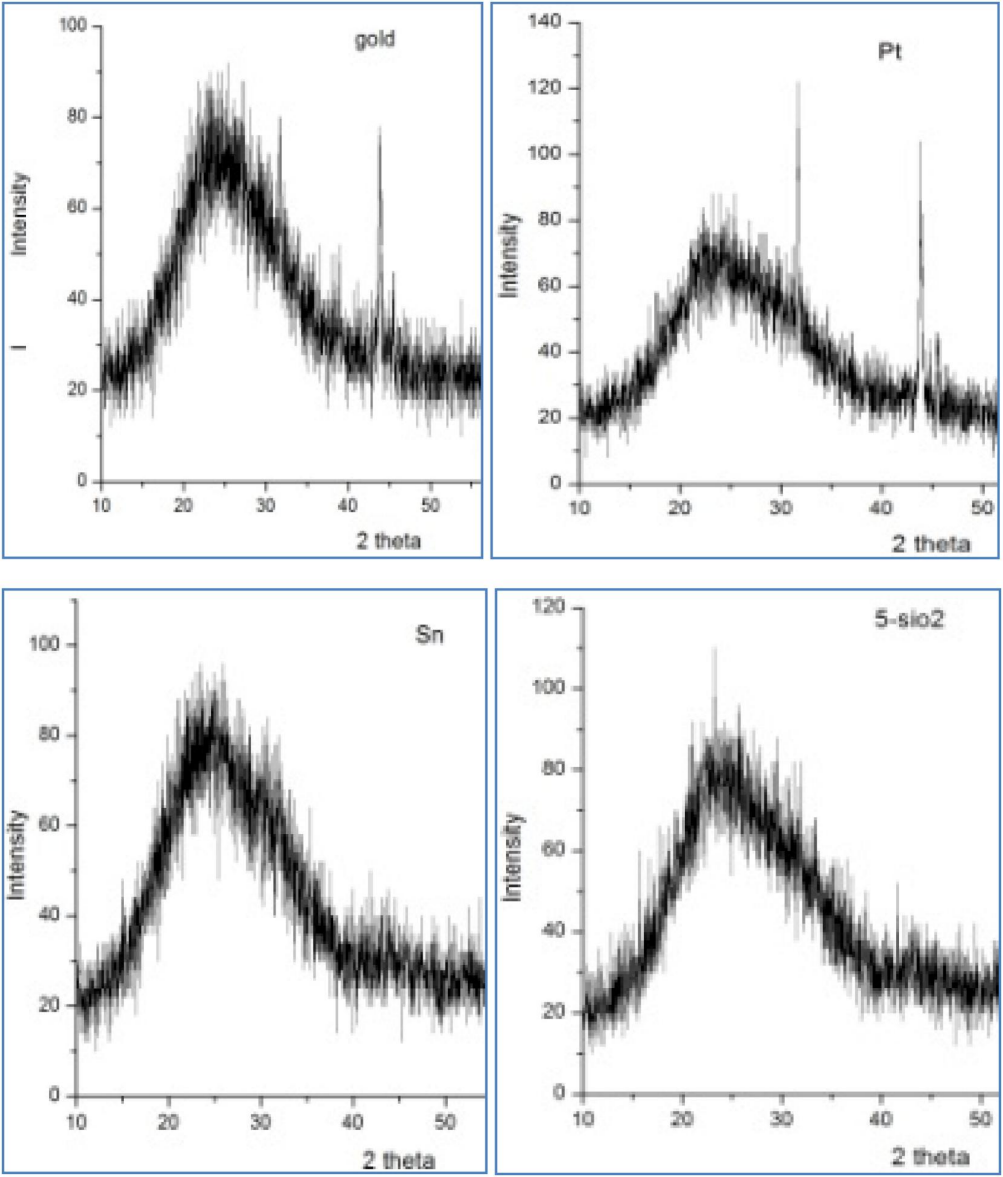
X‐ray diffraction spectrum of Pt, Au, SiO_2_, and SnO_2_ nanoparticles. Au, gold; Pt, platinum; SnO_2_, tin oxide; SiO_2_, silica oxide

The X‐ray diffraction spectrum of the four nanoparticles is shown in Figure 5.

The crystallite size of 200 oriented Au, 111 oriented Pt, 110 oriented SnO_2,_ and 100 oriented SiO_2_ were calculated using the following equation:

$$D=\frac{k\lambda }{\beta \;\cos\;\theta }$$

The results were 13, 28.8, 10.9, and 4.7 nm, respectively.

#### 3.4.3 Transmission electron microscopy investigation

**FIGURE 6:**
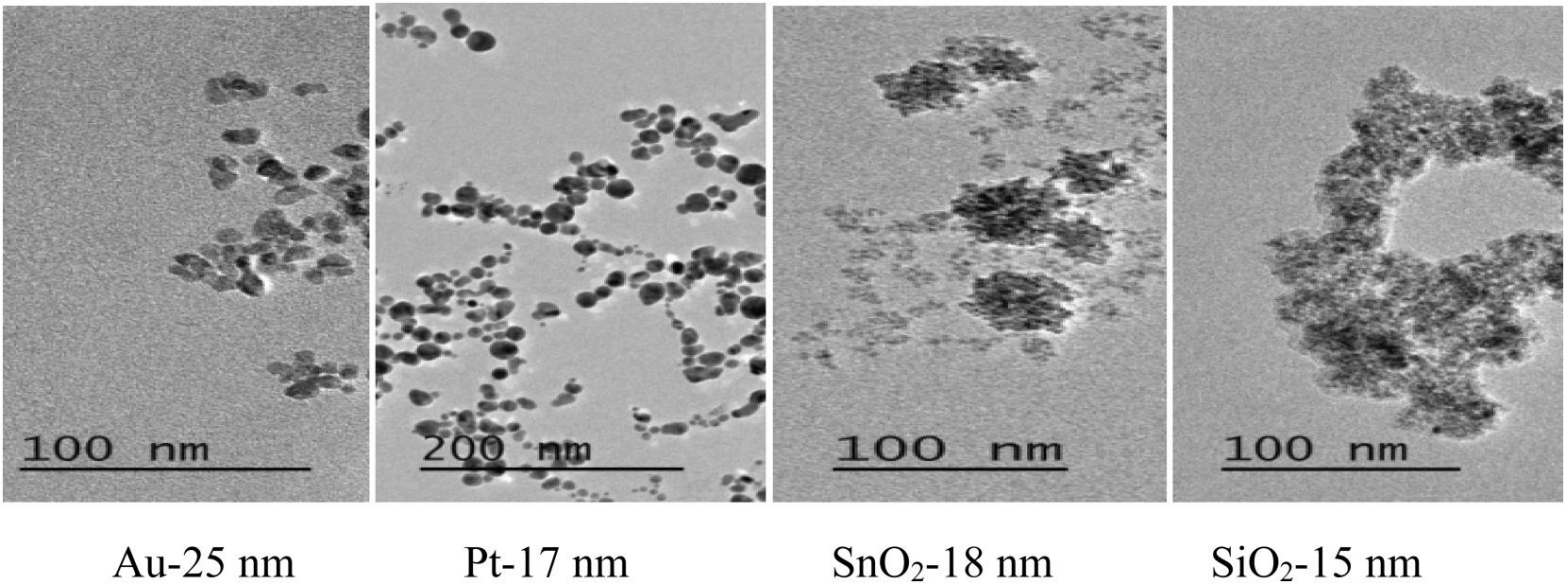
Transmission electron microscopy images of Au, Pt, SnO_2_, and SiO_2_ nanoparticles prepared by laser ablation. Au, gold; Pt, platinum; SnO_2_, tin oxide; SiO_2_, silica oxide

**FIGURE 7:**
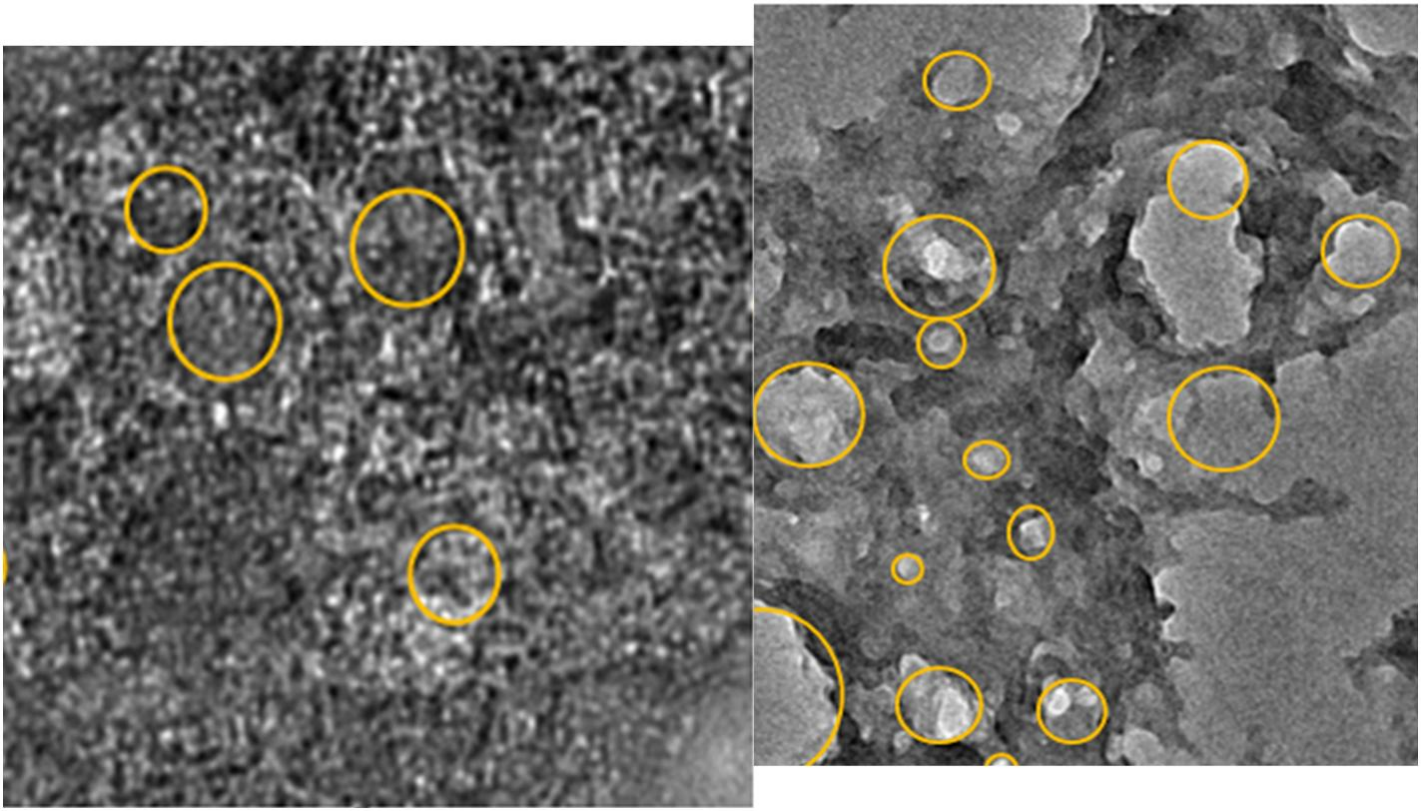
Transmission electron microscopy images of lectin before (on the left) and after (on the right) conjugation with silca oxide nanoparticles

Figure 6 shows TEM images of the prepared nanoparticles. The prepared nanoparticles were spherical, and the average sizes of Pt, Au, SiO_2_, and SnO_2_ nanoparticles were 25, 17, 18, and 15 nm, respectively. Figure 7 shows the TEM image of the produced lectin and the TEM image of SiO_2_‐conjugated lectin_._

### 3.5 Formation of the biofilm and biofilm inhibition

**FIGURE 8:**
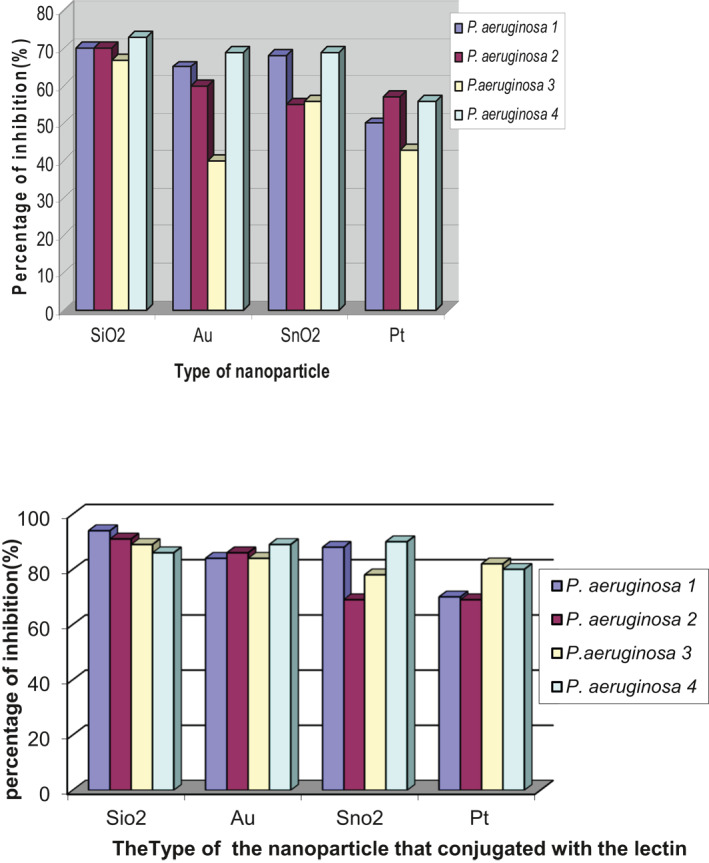
Top: percentage of biofilm formation inhibition by various types of nanoparticles as measured by the polystyrene plate method. Bottom: percentage of biofilm formation inhibition by nanoparticles conjugated with purified lectin as measured by the polystyrene plate method. 1

**FIGURE 9:**
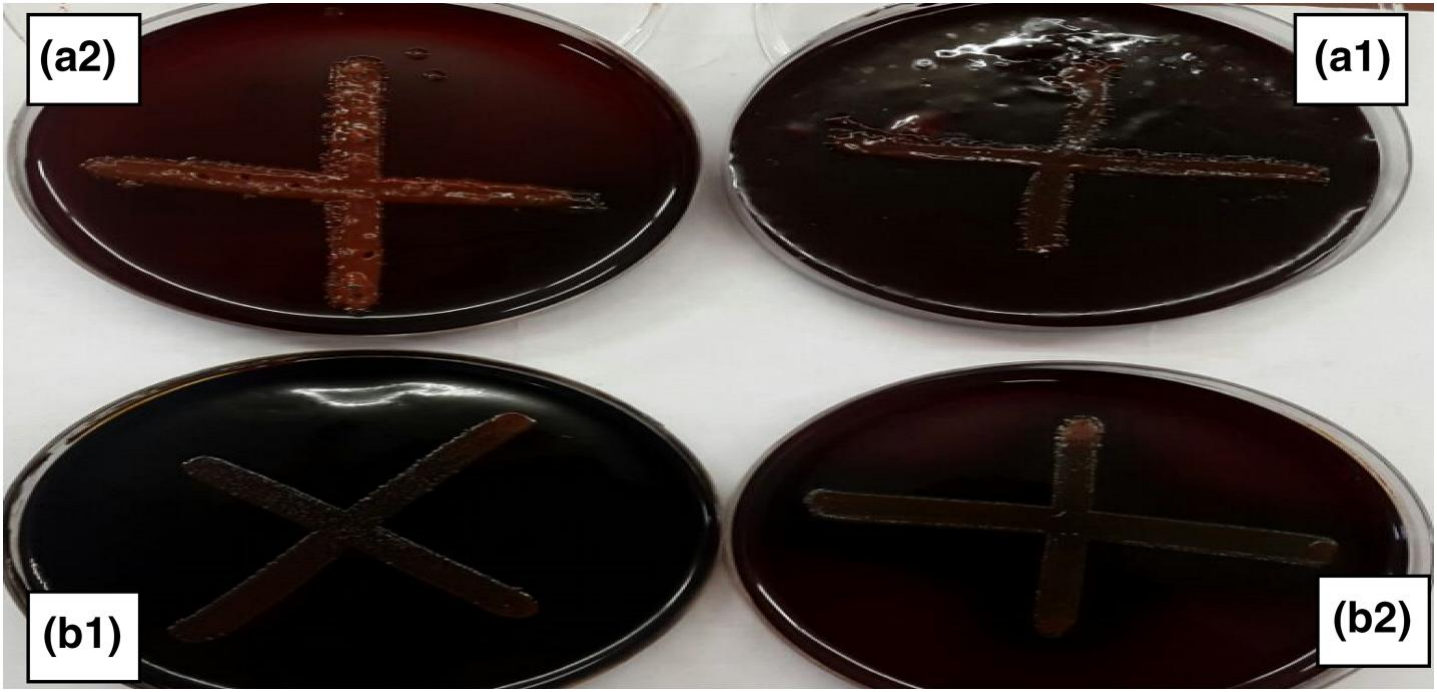
Biofilm formation by the Congo red agar method. Before (a1) and after (a2) addition of purified lectin conjugated with Sio_2_ nanoparticles. Before (b1) and after (b2) addition of purified lectin conjugated with Au nanoparticles

Of 13 *P. aeruginosa* isolates, only four (about 30.8%) produced different capabilities to form a biofilm. The anti‐biofilm activity of the purified lectin (1.01 ng/ml) was only about 70%. On the other hand, the anti‐biofilm activity of the nanoparticles separately, as shown in Figure 8, did not exceed 72%. To evaluate the anti‐biofilm activity of the purified lectin (1.01 ng/ml) with each type of nanoparticle, the four *P. aeruginosa* that formed biofilm were also used. The presence of lectin with SiO_2_ and Au separately led to inhibition of biofilm formation (Figure 9) by the CRA plate. In the polystyrene plate, biofilm inhibition was 94% followed by 91%, 89%, and 86%, respectively, as depicted in Figure 8.

SiO_2_‐conjugated lectin significantly (*p* < 0.05) inhibits biofilm formation compared with results from control and other nanoparticle‐conjugated lectins (Table 2). On the other hand, Au‐conjugated, Pt nanoparticle‐conjugated, and SnO_2_‐conjugated lectins also inhibited biofilm significantly compared with control (*p* < 0.05). Considering these results, SiO_2_‐conjugated lectin was chosen for the gene expression experiments.

**TABLE 2:** Optical density of *Pseudomonas aeruginosa* biofilm treated with nanoparticle‐conjugated lectin

Isolate number	Control	Gold nanoparticle‐conjugated lectin	Platinum nanoparticle‐conjugated lectin	Tin oxide nanoparticle‐conjugated lectin	Silica oxide nanoparticle‐conjugated lectin
	Mean of OD ± SD	Mean of OD ± SD	Mean of OD ± SD	Mean of OD ± SD	Mean of OD ± SD
*P. aeruginosa* 1	0.86 ± 0.28	0.14 ± 0.054*	0.26 ± 0.33*	0.1 ± 0.004*	0.05 ± 0.00**
*P. aeruginosa* 2	0.48 ± 0.1	0.069 ± 0.017*	0.148 ± 0.44*	0.15 ± 0.046*	0.041 ± 0.009*
*P. aeruginosa* 3	0.56 ± 0.02	0.09 ± 0.004*	0.1 ± 0.49*	0.123 ± 0.019*	0.06 ± 0.01*
*P. aeruginosa* 4	0.42 ± 0.1	0.045 ± 0.041*	0.083 ± 0.50*	0.044 ± 0.06*	0.056 ± 0.006*

Abbreviations: OD, optical density; SD, standard deviation.

*p < 0.05 as comparison with control. **p < 0.05 as a comparison with the gold, platinum, and tin oxide groups.

### 3.6 Expression of quorum‐sensing regulatory gene in the presence of lectin and silca oxide nanoparticles

The expression of the *rhlR* gene was determined after and before treatment with lectin and SiO_2_ nanoparticles. The expression of the *rhlR* gene was decreased to 5.26‐fold (Table 3).

**TABLE 3:** Expression fold of the *rhlR* gene after silca oxide–conjugated lectin treatment

SiO_2_‐conjugated lectin treatment	Means of CT of target gene	Means of CT of the housekeeping gene	Δ CT(mean CT of target gene–mean CT of housekeeping gene)	ΔΔCT	2 ^–ΔCT^	Fold of decreasing gene expression
After treatment	27.98	20.27	7.71	7.71‐7.33 = 0.38	0.38	5.26
Before treatment	27.60	20.27	7.33			

Abbreviation: CT, threshold cycle.

## 4 DISCUSSION

About a quarter of the samples of the chicken meat are contaminated with *P. aeruginosa* isolates in the current study. The *Pseudomonas* species are one of the causes of food decomposition [10, 27, 28]. Hang'ombe et al. [29] found that many bacterial species in chicken carcasses—*Salmonella* spp*, Escherichia coli*, *Klebsiella* spp*, Pseudomonas* spp*,* and others—are contaminants of chicken meat. The odours that are mostly associated with spoiled poultry, such as discharge odours, were produced by *Pseudomonas* spp [30]. Kanatt and Chawla [31] also revealed that *Pseudomonas* is the predominant spoilage bacteria found in fresh broiler chicken.

*P. aeruginosa* isolates can produce biofilm as reported here and elsewhere [32]. However, many anti‐biofilm agents have been identified [33, 34, 35, 36]. Recently, nanotechnology has served to identify antimicrobials and anti‐biofilm elements that affect the antibiotic‐resistant bacteria and inhibit biofilm formation [37]. Ag nanoparticles have been used as anti‐quorum sensing agents and as an anti‐ *P. aeruginosa* biofilm [38]. In this study, nanoparticle‐conjugated lectin is used to inhibit biofilm and virulence factor expression as measured phenotypically and genotypically. Marked inhibition in biofilm formation was observed when lectin was conjugated with SiO_2_ and Au separately, leading to inhibition biofilm formation ability by the CRA plate method. In the polystyrene plate method, biofilm inhibition was observed with the four nanoparticles that were conjugated with lectin. This may be the first report of such an effect.

The QS systems contribute to the capability of *P. aeruginosa* to form biofilms on medically main devices [39]. QS plays a central role in biofilm formation by *P. aeruginosa*. Biofilms and QS are affected by the adjacent environment. Nevertheless, numerous associations between biofilm formation and QS have been documented. Two QS systems of *P. aeruginosa* were discovered; they are the N‐acyl‐homoserine lactone–based synthases, Rhl and Las. The Rhl system is composed of the RhlR and *N*‐butyryl‐l‐homoserine lactone [40]. The *rhlR* gene of *P. aeruginosa* has a fundamental effect on QS and encodes for the transcriptional regulator RhlR [41, 42] https://www.ncbi.nlm.nih.gov/pubmed/14600219. It was found that *rhlR* gene expression decreases in the presence of SiO_2_‐conjugated lectin. RhlR regulon regulates a set of virulence factor genes such as elastases, rhamnolipids, and pyocyanin [43]. Conjugated lectins with these types of nanoparticles are potent inhibitors of these virulence factors, and as a consequence, of the virulence of biofilm bacteria.

In conclusion, lectin and Pt, Au, SiO_2_, and SnO_2_ nanoparticles individually are biofilm inhibitors. Pt‐, Au‐, SiO_2‐,_ and SnO_2_‐conjugated lectins are effective biofilm inhibitors, while SiO_2_‐conjugated lectin is the most significant as an anti‐biofilm. Moreover, SiO_2_‐conjugated lectin lowered the levels of virulence factors regulon and RhlR, and as a consequence, reduced the virulence of *P. aeruginosa*.

## Footnotes

1 Au, gold; Pt, platinum; SnO_2_, tin oxide; SiO_2_, silica oxide
